# Impacts of changing work from home patterns on health behaviours and obesity: insights from the late COVID-19 pandemic

**DOI:** 10.1186/s12889-025-25547-2

**Published:** 2025-11-17

**Authors:** Auriba Raza, Paraskevi Peristera, Timo Lanki, Linda L. Magnusson Hanson, Hugo Westerlund, Jaana I. Halonen

**Affiliations:** 1 Division of Psychobiology and Epidemiology, Department of Psychology, Stockholm University, Stockholm, SE-106 91 Sweden; 2 School of Health & Wellbeing, University of Glasgow, Glasgow, G11 6EW United Kingdom; 3 Department of Environmental and Biological Sciences, University of Eastern Finland, Kuopio, FI-70210 Finland; 4 Department of Public Health, Finnish Institute for Health and Welfare, Kuopio, FI- 70701 Finland; 5 Institute of Public Health and Clinical Nutrition, University of Eastern Finland, Kuopio, FI-70210 Finland; 6 Department of Public Health, Finnish Institute for Health and Welfare, P.O. Box 30, Helsinki, FI-00271 Finland

**Keywords:** Remote work, Physical inactivity, Alcohol drinking, Obesity, COVID-19 pandemic, Health behaviour

## Abstract

**Background:**

Numerous studies on work from home during the Covid-19 pandemic link it to reduced physical activity, increased alcohol use, and weight gain, mainly under stringent pandemic restrictions. We investigated whether changes in work-from-home levels from pre to late pandemic are associated with health behaviours during the late pandemic, controlling for family and work factors.

**Methods:**

Using 8195 participants from the 2022 wave of the Swedish Longitudinal Survey of Health, we used logistic regression to analyze the associations between changes in the amount of remote work from pre-pandemic to late pandemic, and physical inactivity, problem drinking, and obesity. Models were first adjusted for age and sex; then for civil status, having children under the age of 12 years at home, and occupation; and finally for job stress, work-family conflict, and family-work conflict.

**Results:**

Individuals who decreased work from home had 17% higher odds of being physically inactive (fully adjusted model OR: 1.17, 95% CI: 1.00–1.37) compared to those who did not change their amount of work from home. Changes in work from home were not statistically significantly associated with problem drinking or obesity. However, there was a tendency for those who decreased work from home to have higher odds of obesity (OR: 1.08, 95% CI: 0.94–1.24), although the association did not reach statistical significance.

**Conclusions:**

These findings suggest, although the associations were weak, that work from home could offer opportunities for individuals to be more conscious of their health and to engage in healthier behaviours.

## Key points

• Changes in remote work were studied in relation to health behaviours post-pandemic.

• Data came from 8,195 participants in the 2022 Swedish Longitudinal Survey of Health.

• Reduced remote work was linked to increased odds of physical inactivity.

• No significant associations were found with problem drinking or obesity.

• Remote work may support healthier behaviour, particularly physical activity.

## Introduction

The COVID-19 pandemic represented an unprecedented global crisis, prompting governments worldwide to implement a range of strategies to mitigate the spread of the virus. These measures, while varying in restrictions and approaches, shared a common goal of reducing social contact to protect public health. Among the most widely adopted interventions was the transition to work from home for workers whose presence at workplace was not essential, a practice that reshaped workplace norms and individual behaviours. The degree of restrictions differed significantly between countries; some imposed strict lockdowns and mandated remote work, others, such as Sweden, adopted a notably distinct approach, refraining from mandating strict stay-at-home orders, enforcing rigid social-distancing measures, or closing national borders [1]. Many businesses and childcare facilities remained operational, and instead of imposing strict regulations, authorities encouraged people to work from home when possible, placing the emphasis on personal responsibility rather than government mandates. [1–3]. In 2016, before the pandemic, 2% of Swedish employees mainly worked from home, and 13% did so occasionally. [4]. When the pandemic began in 2020, large-scale remote work was implemented with limited organisational experience of its complexities, creating scope for unanticipated challenges. Over time, as the pandemic persisted, working from home became more familiar, and hybrid arrangements increasingly became part of the new normal.

During this time, a surge of studies emerged globally using different study designs and methodologies to study the impact of this “new norm” - working from home – on workers’ health behaviours in abnormal circumstances both at home and work. Most early-pandemic studies linked working from home to lower physical activity and higher sedentary time in settings with higher scores [5–9] on the Oxford COVID-19 Government Response Tracker Stringency Index. This index is a composite measure derived from 23 indicators covering containment and closure policies, testing and vaccination strategies, and health-system measures with a range from 0 to 100; 0 indicates no restrictions and 100 indicates a total lockdown [10]. For example, a longitudinal study from Switzerland, that utilized baseline data from before the pandemic and follow-up data collected during soft COVID lockdown policies, did not observe reductions in physical activity among remote workers [11]. In contrast, a longitudinal study from Thailand, a country with rather strict contingency measures, observed a 50-minute/day increase in sedentary time among remote workers during the pandemic [12]. Studies from settings with lower scores reported mixed findings [11, 13].

The majority of the published literature focused on the physical activity and sedentary behaviour of remote workers, likely due to the well-established positive effects of physical activity on both physical [14]and mental health [15], that were big concerns during the pandemic. Impact of working from home on alcohol consumption and weight gain was also of concern, though comparatively less studied. A longitudinal within-individual study conducted in the first year of the pandemic reported greater alcohol consumption when participants worked from home compared to when they did not work from home [16]. Similar findings were echoed in cross-sectional studies, with increased alcohol consumption among remote workers [17–20]. Noteworthy, in one study working from home was only associated with increased alcohol consumption among individuals who would have preferred not to work from home [21].

As the pandemic has passed, work from home has transitioned from a crisis-driven necessity to an accepted part of the post-pandemic work environment. Lessons learned during the pandemic can help shape the post-pandemic work environment, however, there remains lack of research on the impact of post-pandemic remote work on health behaviours. Studies conducted in the later stages of the pandemic, particularly in countries with minimal or no restrictions, can provide valuable insights into the potential benefits or harms of remote work under more stable and normal conditions.

Sweden’s approach to handling the pandemic, both praised and criticized [3], provided a unique opportunity to examine the impact of work from home on health behaviours in a context where outdoor spaces remained accessible and indoor sports facilities operated with limited capacity throughout the pandemic. As work from home becomes accepted in the post-pandemic work life, studying its impact on individual health behaviours, such as physical activity and alcohol consumption, as well as body mass index (BMI), in this relatively unrestricted environment provides valuable insights for shaping workplace and public health policies, especially given the limited post-pandemic evidence.

Thus, in our study, using data from the 2022 wave of the Swedish Longitudinal Survey of Health (SLOSH), we investigated the impact of changes in the amount of working from home from pre-pandemic to late pandemic on health behaviours and BMI during the late stage of the pandemic controlling for several family- and work-related factors. This period was characterized by the lifting of restrictions in an already restriction-free setting, as people began returning to what were considered normal circumstances.

## Materials and methods

### Study population

The study participants are from a unique longitudinal cohort, the Swedish Longitudinal Survey of Health (SLOSH), which is designed to be nationally representative of the Swedish working population [22]. SLOSH was started in 2006 as a biennial nationally representative survey of work-life participation, work environment, and health and well-being. The original sample included all respondents of the 2003 biennial Work Environment Survey (AMU), which itself was drawn from the annual Labour Force Survey (AKU) administered by the Work Environment Authority and Statistics Sweden [22, 23]. In subsequent years up to 2020, SLOSH was supplemented with respondents from the AMU surveys conducted between 2003 and 2011, and since 2022 additional respondents from the 2013–2019 AMU surveys have also been included [23]. Although attrition has occurred over time, this continuous supplementation has ensured that SLOSH remains broadly representative of the Swedish working population, even in the context of demographic and societal changes since its inception. The SLOSH started as a biennial survey and since 2022 questionnaires are sent annually. Partly different sets of questions are directed at individuals who worked more than 30% of a full-time schedule during the three months preceding the survey, as well as those employed less than 30%, in temporary jobs, or permanently unemployed.

**Fig. 1:**
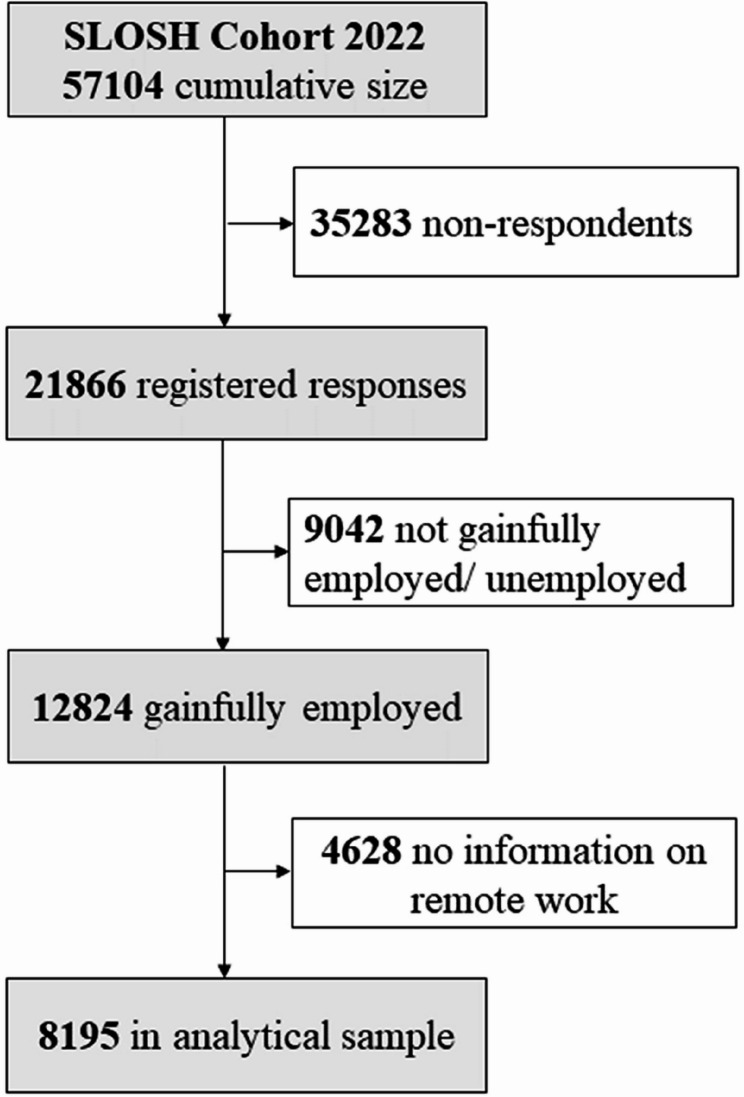
Selection of analytical sample

This study focused on gainfully employed participants from the 2022 SLOSH wave. The cumulative cohort comprised of 57,104 participants. Of these, 21,866 returned the questionnaire (response rate of 42.5%). We excluded respondents who were not gainfully employed (*n* = 9,042) and those with missing information on work from home (*n* = 4,628). The final analytical sample included 8,195 gainfully employed participants with complete data on remote work, physical inactivity, problem drinking, BMI, and the covariates (Fig. 1). The Swedish Ethics Review Authority has approved this study (Dnr: 2022–02640-01). Participants of the Swedish Longitudinal Occupational Survey of Health (SLOSH) received written information on the survey, and return of the survey indicated informed consent to participate.

### Measures

#### Change in work from home

In the survey, participants were asked, “Did you work from home more or less during the past month compared to before the pandemic?” The response options were: (1) definitely more, (2) the same, and (3) definitely less. These responses were categorized as an increase in work from home, the same as before the pandemic, and a decrease in work from home, respectively. In the analyses, participants whose amount of work from home remained unchanged. were used as the reference group.

#### Physical inactivity

Physical inactivity of participants was evaluated by using a single item question “How much do you exercise? including walking and cycling to and from work” with response alternatives: never exercise, move very little or take occasional walks, exercise now and then, and exercise regularly. Participants were categorized as physically inactive if they selected the first or second response alternative and active if they selected the third or the last alternative [24].

#### Problem drinking

Problem drinking was assessed with amodified Cut-Annoyed-Guilty-Eye questionnaire (CAGE) [25]. The modification to the original questionnaire was that the word “ever” was removed from the questions to avoid making them questions about lifetime problems. CAGE is a brief, validated screening tool for alcohol-related problems in clinical and population settings, with reported sensitivity of 93% and 76% specificity for identifying problem drinking and the score of two or more is considered clinically significant problem [26]. The questionnaire included the following questions for participants reporting any drinking; (1) Have you felt you should cut down on your drinking? (2) Have people annoyed you by criticizing your drinking? (3) Have you felt bad or guilty about your drinking? (4) Have you had a drink first thing in the morning to steady your nerves or get rid of a hangover? Consistent with standard practice, a score of two or more affirmative responses was coded as a positive CAGE screen, hereafter referred to as problem drinking. [27]. This operationalisation captures consequences of and loss of control related to alcohol drinking rather than consumption volume, which aligns with our focus on harmful use. We acknowledge that CAGE is a screen and not a diagnostic instrument, and it does not quantify intake.

#### Obesity

BMI was calculated based on self-reported weight in kilograms (kg) divided by self-reported height in meters squared in each follow-up. Obesity variable was dichotomized as: obese BMI ≥ 30 & <50 vs. not obese BMI > = 14 & <30 [24], thus excluding individuals with extreme values.

#### Covariates

To control for possible confounders, we selected a set of covariates based on prior studies [11–13, 28]. Sex (men and women) and age (categorized as 18–29, 30–39, 40–49, 50–59, ≥ 60 years) were obtained from registers. Family-related variables, civil status (married/cohabiting vs. not), children at home under the age of 12 years at home (yes or no), and occupation were self-reported. Statistics Sweden, using self-reported information, coded the occupations according to a socioeconomic classification which was then used for categorization of occupational positions into low, intermediate, high, and self-employed [29]. Occupation was considered a family-related factor because it is used as a proxy for the socioeconomic position of the participants and their households rather than specific work characteristics.

Job strain was assessed based on the Swedish Demand Control Questionnaire including five job demand items; (1) working fast, (2) working hard/intensively, (3) no excessive amount of work/too much effort, (4) enough time, and (5) conflicting demands, and six job control items; (1) learn new things, (2) high level of skill, (3) creativity/initiative, (4) repetitive work, (5) a lot of say/what to do, and (6) little freedom/how to do [22]. Means of the five job demand and six job control items were calculated. Participants with mean demands scores above the median and mean control scores below the median were categorized as having job strain [30]. Work-family conflict and family-work conflict were assessed using a questionnaire originally developed by Fisher et al. (2009) [31]and adapted for use in Swedish. Work-family conflict was evaluated through four statements: (1) I come home too tired to do things I would like to do, (2) My job makes it difficult to maintain the kind of personal life I would like, (3) I often neglect my personal needs because of the demands of my work, (4) My personal life suffers because of my work. While three statements measured family-work conflict; (1) My work suffers because of everything going on in my personal life, (2) I would devote more time to work if it weren’t for everything I have going on in my personal life, (3) I am too tired to be effective at work because of things I have going on in my personal life. Responses were recorded on a five-point Likert scale ranging from “not at all” to “almost always.” Two conflict scores were calculated as the mean of the respective items, with higher scores indicating greater levels of conflict [32].

### Statistical analyses

We have used logistic regression to analyze the associations between changes in the amount of work from home, comparing the level during the late pandemic with the pre-pandemic level, and physical inactivity, problem drinking, and obesity. We adjusted for potential confounding sequentially. First, we accounted for age and sex in the models. Second, we included family-related factors, i.e., civil status, children under the age of 12 years at home, and occupation. Finally, in the full model, we incorporated the work-related factors: job stress, work-family conflict, and family-work conflict. To evaluate possible effect modification, interaction terms for the aforementioned covariates with work from home were included in the full model.

As a sensitivity analysis, we modelled BMI as a continuous variable using a generalized linear model (PROC GENMOD) with a normal distribution to increase statistical power and to assess the robustness of our main findings. BMI values were restricted to the range of 14–50 kg/m² to exclude implausible values. We were unable to treat physical inactivity and problem drinking as continuous variables due to the categorical and ordinal nature of their original measures.

Effect estimates are presented as odds ratios (OR) with 95% confidence intervals (CI), assessing odds among groups with changes in work from home compared to the reference group, who had no change in the amount of work from home. All analyses were performed using SAS 9.4. Study method and results are reported following the Strengthening the Reporting of Observational Studies in Epidemiology (STROBE) Statement for cross-sectional studies [33].

## Results

In our analytical sample, 55% were women, 58% cohabiting, and 39% belonged to the age group 50–59 years old (Table 1). High occupational positions were more common in the analytical sample (43%), while low occupational positions were more frequent among the excluded (46%) (Table 2). This difference was likely explained by the low positions missing the status of work from home, i.e., they less often had the option to work from home. Nearly half of the analytical sample had children under the age of 12 years at home, and the prevalence of job strain was low (Table 1). In terms of the main variables of interest, 38% of participants reported no change in the amount of work from home during the pandemic compared to pre-pandemic levels. Physical inactivity (16%) and obesity (21%) were more common than problem drinking (6%) at the time of the survey (Table 1).

**Table 1:** Characteristics of the study participants eligible for the analysis (*n* = 8,195)

Variables	Change in the amount of work from home		
	No change	Decrease	Increase
**N (%)**	3104 (38)	2162 (26)	2929 (36)
**Outcomes**			
Physically inactive	367 (17)	475 (15)	463 (16)
Problem drinking	134 (6)	189 (6)	176 (6)
Obesity	494 (23)	671 (22)	569 (19)
**Covariates**			
Women	1308 (60)	1491 (48)	1313 (45)
Cohabiting	1229 (57)	1811 (58)	1719 (59)
Age, years			
18–29	18 (1)	24 (1)	26 (1)
30–39	173 (8)	294 (10)	347 (12)
40–49	525 (24)	702 (23)	862 (29)
50–59	885 (41)	1220 (39)	1097 (38)
≥ 60	561 (26)	863 (28)	597 (20)
Occupational position			
Low	179 (8)	339 (11)	43 (2)
Intermediate	1041 (48)	1493 (48)	1386 (47)
High	904 (42)	1162 (37)	1455 (50)
Self-employed	2 (1)	43 (1)	1 (0.03)
Children under the age of 12 years at home	987 (46)	1439 (46)	1572 (54)
Job strain	296 (14)	349 (11)	347 (12)

**Table 2.** Characteristics of excluded participants (n=4629) in comparison with included sample (n=8,195)

Variables	Included individuals	Excluded individual *N* (%)
**Change in the amount of work from home**		
No change	3104 (38)	NA
Decrease	2162 (26)	NA
Increase	2929 (36)	NA
**Outcomes**		
Physically inactive	1305 (16)	880 (19)
Excessive alcohol use	499 (6)	261 (6)
Obesity	1734 (21)	1177 (25)
**Covariates**		
Women	4537 (55)	2745 (59)
Cohabiting	4759 (58)	2459 (53)
Age, years		
18–29	68 (1)	44 (1)
30–39	814 (10)	397 (9)
40–49	2089 (26)	773 (17)
50–59	3202 (39)	1716 (37)
≥ 60	2021 (25)	1691 (37)
Occupational position		
Low	561 (7)	2136 (46)
Intermediate	3920 (48)	1948 (42)
High	3521 (43)	453 (10)
Self-employed	46 (1)	26 (1)
Children under the age of 12 years at home	3998 (49)	1583 (34)
Job strain	992 (12)	865 (19)

Compared with no change, decrease in work from home was associated with higher odds of physical inactivity in the age- and sex-adjusted model, but the association was not statistically significant (OR 1.15, 95% CI 0.99–1.34). The association reached significance when family-related factors were added (OR 1.18, 95% CI 1.01–1.37; *n* = 8,000) and remained of similar magnitude after further adjustment for work-related factors (OR 1.17, 95% CI 1.00–1.37; *n* = 7,803; Table 3). Also, participants who decreased their amount of work from home during the pandemic indicated higher odds of obesity (OR 1.08, 0.94–1.24) compared with those whose work from home level remained the same, but the association was not statistically significant (Table 3). Results from the sensitivity analysis, where BMI was modelled as a continuous outcome, confirmed these findings. Participants who decreased their work from home had a higher mean BMI (β = 0.40, 95% CI 0.16–0.65) compared with those with no change, while those who increased their remote work showed a smaller, non-significant difference (β = 0.15, 95% CI − 0.07–0.37).

**Table 3:** Association of decreases and increases in work from home (during the late pandemic compared to pre-pandemic period) with health-related behaviours and obesity, compared to individuals with no change in the amount of work from home

Outcomes/Adjustments	No. of obs	Changes in the amount of work from home		
		No change	Decrease	Increase
			OR (95% CIs)	OR (95% CIs)
Physical inactivity				
Age and sex	8158	1	1.15 (0.99–1.34)	1.05 (0.91–1.21)
+ family related factors^a^	8000	1	1.18 (1.01–1.37)	1.12 (0.97–1.30)
+ work related factors^b^	7803	1	1.17 (1.00–1.37.00.37)	1.11 (0.96–1.29)
Excessive alcohol use				
Age and sex	7136	1	1.06 (0.84–1.34)	1.00 (0.81–1.24)
+ family related factors	6992	1	1.05 (0.83–1.32)	0.99 (0.80–1.24)
+ work related factors	6815	1	1.05 (0.83–1.34)	0.97 (0.77–1.21)
Obesity				
Age and sex	8051	1	1.08 (0.94–1.23)	0.90 (0.79–1.02)
+ family related factors	7896	1	1.10 (0.96–1.25)	0.96 (0.84–1.09)
+ work related factors	7702	1	1.08 (0.94–1.24)	0.96 (0.84–1.09)

^a^ cohabiting, occupational position, and children under the age of 12 years at home

^b^ Job strain, work family conflict, and family work conflict

No effect modification was observed by age, sex, civil status, children under the age of 12 yearsat home, occupational position, job strain, work-family conflict and family-work conflict with physical inactivity, age, problem drinking, and BMI (all p-values for interactions > 0.05).

## Discussion

We investigated the associations of an increase or decrease in the amount of work from home from pre-pandemic to late pandemic, with physical inactivity, problem drinking, and obesity using participants whose amount of work from home remained unchanged as a reference. In our results, we observed higher odds of physical inactivity levels among individuals who decreased their work from home compared to the pre-pandemic levels. Supporting this observation, the sensitivity analysis using BMI as a continuous outcome showed a statistically significant higher mean BMI among those who reported a decrease in remote work. No statistically significant associations were observed between changes in work from home and problem drinking, nor was effect modification evident by age, sex, civil status, occupational position, job strain, or other covariates.

Our results are not consistent with the existing literature in which studies have observed significant decreases in physical activity among individuals who worked from home during the pandemic [5–9, 28, 34]. However, comparison to other studies is not straightforward as our focus was on how changes in the extent of work from home are related to health behaviours, rather than work from home per se. Our results align with a study from Switzerland that did not observe statistical difference in the levels of physical activity before the pandemic and during first COVID lockdown [11]. During lockdowns, Switzerland closed indoor activity facilities, but allowed outdoor recreational activities with safety measures, while in Sweden, there were no restrictions on being outdoors while maintaining distance was recommended and indoor activity facilities also remained open with limited capacity and thus provided people opportunities to remain physically active. This could be one of the explanations for the unexpected results. Another likely explanation might be that during the pandemic people became more conscious of their physical and mental health and with the absence of physical activity associated with commuting to work, individuals who were working from home might have engaged in an intentional physical activity to compensate for this change and to remain healthy. This reasoning is supported by National Public Health Survey 2022, conducted by Public Health Agency of Sweden, in which 68% of the Swedish population reported being physically active for at least 150 min per week [35]. Another plausible explanation is that, with more time spent at home and the time saved from commuting, people had more time to engage in physical activities particularly outdoors, even on workdays. A study from Sweden supports this explanation as it reported increased visits to green areas during the pandemic, and more visits to green areas for physical activity when people spent more time at home [2]. Finally, our results demonstrate the importance of adequate confounder control: the association between reduced working from home and physical inactivity became statistically significant only after family-related factors were included as covariates. However, we did not observe effect modification by any of these family-related factors.

In this study, we did not observe a statistically significant difference in problem drinking among participants who increased or decreased their amount of remote work during the late pandemic compared to those whose remote work remained unchanged. Several studies published during the pandemic observed increased alcohol consumption among individuals working from home[16–18, 20]. Specifically, increased alcohol consumption was observed among those who were home-schooling their children compared to the time when there were not [16], as well as among individuals experiencing psychological distress [27]. Our findings should be interpreted in the context of a setting where pre-schools and schools remained open, people were free to move outdoors, indoor leisure facilities and sports training facilities for both children and adults remained open with limited access, and work from home was recommended but not mandatory [1, 3]. Prior research has observed higher alcohol dependence among those under lockdowns compared to those not under restrictions [19], as well as increased alcohol consumption related to work from home among individuals who did not prefer working from home [21].

Our study has several limitations. First, the responses to the SLOSH survey were collected from late spring to late summer. In Sweden, during this time of year people usually engage in outdoor activities, which may introduce desirability bias, as participants might report healthier behaviours than they exhibit during winter. As no responses from winter are available, the effects of seasonality cannot be examined in detail, and, since exact response dates were unavailable, potential seasonal variation between months could not be accounted for. Second, physical activity was measured with a single-item question that did not capture frequency, duration, or intensity. This simplified measure might misclassify physically activity and could have resulted in overestimating activity levels, thereby diluting the associations observed for physical inactivity. Third, our measure of problem drinking is a CAGE screen rather than a diagnostic instrument, and it does not quantify alcohol intake. This may limit comparability with studies that use quantity–frequency definitions. Our measure may misclassify some individuals by capturing perceived consequences or feelings of guilt without high consumption, or by missing hazardous consumption among those without such consequences or feelings. However, any resulting misclassification is likely non-differential with respect to the study exposures, which would bias estimates towards the null. Fourth, while we examined changes in work from home between pre-pandemic and late-pandemic time, the data were collected at a single time point, relying on retrospective self-reporting and one reporting time point for health behaviours. This limits the ability to draw causal conclusions about changes in behaviours. Fifth is the absence of pre-pandemic measures of health and health behaviours, including self-rated health, chronic illness, BMI, physical activity, and alcohol use. This prevents adjustment for health-related selection into working from home and may leave residual confounding that could bias associations in either direction. Sixth, the analytical sample comprised a higher proportion of participants in intermediate and high occupational positions who were more likely to have the option to work from home. While this reflects the target population for whom remote work was feasible, it should be acknowledged that the findings may not fully represent workers in occupations without such flexibility. Lastly, all outcome variables were self-reported, which may lead to underreporting of unhealthy behaviours and might introduce bias.

A key strength of this study is the use of data from SLOSH, which is based on a nationally representative sample of the Swedish working population, enhancing the generalizability of the findings to this population. In addition, the study’s timing, during the late stage of the pandemic when most restrictions had been lifted, provides valuable insights into health behaviours in a more stable and voluntary remote work context, in contrast to crisis-driven conditions in earlier phases. Sweden’s unique pandemic strategy, which emphasized personal responsibility and avoided strict lockdowns, further offers a distinct policy environment to investigate remote work in a setting with minimal external constraints. Finally, the study accounts for a wide range of potential confounding factors, including sociodemographic characteristics, job strain, and work-family conflict, which strengthens the internal validity of the findings.

## Conclusion

A decrease in the amount of work from home during the late pandemic compared to pre-pandemic period was associated with higher odds of physical inactivity. For obesity, the association was positive but not statistically significant; however, in the sensitivity analysis where BMI was treated as a continuous outcome, participants who decreased their remote work had significantly higher BMI. However, no statistically significant associations were observed with problem drinking. Our findings may suggest that maintaining working from home could support healthy behaviours, although further research is needed to explore the underlying mechanisms. Together with other health promotion aspects, these results can be considered when revising workplace policies to support healthy habits, ensuring that the benefits of work flexibility are maximized.

## Data Availability

The datasets generated and/or analysed during the current study are not publicly available due to ethical issues. However, we agree to allow the journal to review our data if requested, given that such a request can be granted by Stockholm University based on relevant legislation at the time of the request. Corresponding author should be contacted for the data requests.
